# A Two-Stage Distributed Task Assignment Algorithm Based on Contract Net Protocol for Multi-UAV Cooperative Reconnaissance Task Reassignment in Dynamic Environments

**DOI:** 10.3390/s23187980

**Published:** 2023-09-20

**Authors:** Gang Wang, Xiao Lv, Xiaohu Yan

**Affiliations:** 1 College of Computer Engineering, Naval University of Engineering, Wuhan 430033, China; mingyue_nue@163.com; 2 School of Undergraduate Education, Shenzhen Polytechnic University, Shenzhen 518055, China; yanxiaohu@szpt.edu.cn

**Keywords:** multi-UAVs, task reassignment, distributed algorithm, two stage, contract net protocol

## Abstract

Multi-UAV systems have been widely used in reconnaissance, disaster relief, communication, and other fields. However, many dynamic events can cause a partial failure of the original mission during the mission execution process, in which case task reassignment should be carried out. How to reassign resources and tasks in multi-dynamic, multi-target, and multi-constraint events becomes a core issue in the enhancement of combat efficiency. This paper establishes a model of multi-UAV cooperative reconnaissance task reassignment that comprehensively considers various dynamic factors such as UAV performance differences, size of target areas, and time window constraints. Then, a two-stage distributed task assignment algorithm (TS-DTA) is presented to achieve multi-task reassignment in dynamic environments. Finally, this paper verifies the effectiveness of the TS-DTA algorithm through simulation experiments and analyzes its performance through comparative experiments. The experimental results show that the TS-DTA algorithm can efficiently solve the task reassignment problem in dynamic environments while effectively reducing the communication burden of UAV formations.

## 1. Introduction

Compared with manned aircrafts, UAVs have the advantages of low cost, strong concealment, and being unmanned [1,2,3]. They are widely used in reconnaissance, disaster relief, communication, and other fields [4,5,6,7]. However, with the continuous expansion of task scales, in many cases, a single UAV cannot complete its tasks efficiently [8]. Multi-UAV systems have higher efficiency and stronger robustness than single UAVs [9,10]. In order to improve the efficiency and success rate of reconnaissance tasks, multi-UAVs often perform them cooperatively, especially in the field of military reconnaissance. Task assignment is a key issue for multi-UAVs to cooperate efficiently [11].

Before a reconnaissance mission begins, multi-UAV systems need to assign tasks according to static prior information and obtain the initial mission plan. In recent years, many researchers have established models and proposed solutions to such task assignment problems [12,13,14,15,16]. However, there are many uncertainties in the actual task execution process, such as target movement, drone damage, and many other dynamic events [17]. When these dynamic events occur, multi-UAV systems need to adjust the initial mission plan to adapt to these changes, a process called task reassignment [18].

Many researchers have established relevant models of task reassignment in dynamic environments. Yang et al. [17] established a task reassignment model for multi-UAVs performing search and rescue (SAR) missions, considering five dynamic events: finding new tasks, canceling original tasks, updating task coordinates, updating task deadlines, and updating task durations. However, the model only involved dynamic changes of targets and did not consider damage to UAVs during the mission. In the context of multi-UAV cooperative task assignment in SAR missions, Chen et al. [19] considered time sensitivity and dynamic environments and studied the problem of multi-UAV task reassignment after the emergence of a new target. However, their model did not consider the simultaneous occurrence of multiple targets. Oh et al. [20], in the context of suppression of enemy air defense (SEAD) missions, considered the problem of task assignment in the case of pop-up threats and UAV loss. However, the performance differences of UAVs were not considered in the model. Zhang et al. [21] studied the dynamic task assignment problem in the context of multi-UAVs attacking multiple ground targets cooperatively and considered the emergence of new targets and sudden UAV failure. However, the time window constraint of targets and movement of targets were not considered in the model.

Due to the nature of armed conflict, multi-UAVs are often in a dynamic environment when performing a cooperative reconnaissance mission. Existing multi-UAV task reassignment models in dynamic environments are not directly used for the modeling of multi-dynamic, multi-target, and multi-constraint events. In order to better describe the problem of multi-UAV cooperative reconnaissance task reassignment and discuss the literature on the above subject, this paper establishes a model of multi-UAV cooperative reconnaissance task reassignment in dynamic environments (DE-MCRTR) based on our previous work [22]. The DE-MCRTR model addresses task reassignment problems in dynamic events, including UAV damage, new target occurrence, location change of the target, as well as time window change of the target, while comprehensively considering various factors such as UAV performance differences, size of target areas, and time window constraints.

Multi-UAV task assignment is an NP-hard problem [23,24]. Commonly used task assignment algorithms can be divided into two categories [5,19]: one comprises centralized algorithms and the other comprises distributed algorithms. Centralized algorithms have the ability of global optimization [24] but their computational demand is high [17]. So, the solution speed of centralized algorithms is low, which causes their slow responsiveness to dynamic changes [25]. Although distributed algorithms cannot obtain global optimal solutions [24], their computational complexity is relatively small and their stability is higher [26]. Since task reassignment in dynamic environments is carried out in the process of mission execution, higher requirements are placed on the speed of reassignment. Thus, researchers usually use distributed task assignment algorithms to solve the problem of task reassignment in dynamic environments. The most common are consensus-based auction algorithms and market-based algorithms [27]. Among them, the most widely used are consensus-based bundle algorithms (CBBAs) [28] and the contract net protocol (CNP) [29].

Aiming at the problem of UAV swarms performing cooperative reconnaissance-and-attack tasks on multiple targets in complex and uncertain combat scenarios, Qin et al. [30] proposed a cooperator determination mechanism and a selection mechanism of sequential tasks, and extended the contract net protocol to design an event-triggered dynamic task allocation strategy. This strategy adopted the selection mechanism of sequential tasks and assigned tasks one by one. When the number of tasks was large, it was difficult to ensure efficiency. Zitouni et al. [31] proposed a distributed multi-UAV task assignment algorithm by combining a consensus-based bundle algorithm and an ant colony algorithm. However, the algorithm was only applicable if some original tasks were cancelled. Using the state feedback, gradient descent, and primal–dual methods, Deng et al. [32] designed a distributed algorithm for high-order agents to perform resource allocation tasks autonomously. Zhang et al. [33] introduced a sales contract, exchange contract, and replacement contract into the original contract net protocol and proposed an algorithm based on a hybrid contract net protocol to achieve dynamic task reassignment. This method had a low proportion of feasible solution combinations in the solving process; so, the solving efficiency was low when the number of targets is large. Yang et al. [17] proposed a partial reassignment algorithm based on the PI algorithm to realize online task reassignment after the target dynamic changes during mission execution. The algorithm was used for a single task reassignment situation, and it was not suitable for multiple tasks. Gao et al. [34] designed a task reassignment algorithm based on the contract net protocol to solve the task reassignment problem when new targets appear or UAV damage occurs during task execution. However, with the expansion of the scale of the problem, the number of UAV communications would be multiplied, resulting in poor reliability of the system.

There are multiple targets that need to be assigned in the DE-MCRTR model, and some targets contain time window constraints. In order to realize rapid reassignment of multiple targets, reduce the communication burden of multi-UAV formation, and ensure the quality of task assignment to a certain extent, this paper proposes a two-stage distributed task assignment algorithm based on contract net protocol (TS-DTA). The goal of the first stage and the second stage of the TS-DTA algorithm is to assign the targets without a time window and the targets with a time window, respectively. Based on the original contract net protocol, the bidding strategy with bidding benchmark is introduced in the first stage to reduce the communication burden of UAV formation, the bidding strategy based on route distance is proposed in the first stage to realize synchronous assignment of multiple tasks, and the cyclic bidding strategy based on task timing is proposed in the second stage to achieve efficient assignment of targets with time window constraints.

The rest of this paper is organized as follows: In Section 2, the background of multi-UAV cooperative reconnaissance task reassignment problem is introduced, and the task reassignment model is established. In Section 3, the TS-DTA algorithm is introduced in detail. In Section 4, a series of simulation experiments is conducted. In Section 5, the conclusions and prospects are given.

## 2. Task Reassignment Model

This section first introduces the background of the task reassignment problem of multi-UAV cooperative reconnaissance, and then establishes the task reassignment model according to dynamic events.

### 2.1. Background

The reconnaissance in this paper refers to using the photoelectric equipment and radar carried by UAVs to obtain real-time video and image information of targets so as to provide information support for decision-making for the command center. After the initial mission plan is obtained based on static prior information, UAVs start from their ship platform to each target to perform reconnaissance. The circular reconnaissance route is adopted by UAVs when performing reconnaissance. The radius of circular route is dependent on the danger degree and the size of each target. After completing all tasks, UAVs return to the ship platform. However, in the actual mission execution process, the confrontation between the enemy and ourselves would lead to many dynamic events, such as damage to the UAV, movement of targets, and so on. These dynamic events may partially invalidate the original mission plan. In order to ensure the completion of all tasks, it is necessary to reasonably assign unfinished tasks to currently dispatched UAVs. Combined with the actual mission, this paper focuses on the following four kinds of dynamic events.

(1) UAV damage

During the mission, a drone is suddenly hit by enemy air defense forces, making it impossible to complete the remaining tasks.

(2) New target occurrence

During the reconnaissance mission, early warning aircraft or ships may find some new targets that need to be reconnoitered.

(3) Location change of target

In order to enable UAV formation to quickly capture the moving targets, the provider of prior information needs to update the location of those targets in time.

(4) Time window change of target

In order to realize an effective cooperation with other combat forces, UAVs need to reconnoiter some targets within a given time window. When the time window of some targets are advanced or pushed back, the original plan may no longer meet the requirements of the new time window.

### 2.2. DE-MCRTR Model

Based on the previous work [22], this article introduces dynamic events and establishes a multi-UAV cooperative reconnaissance task reassignment model in a dynamic environment. The specific model is as follows:

#### 2.2.1. Basic Information of the Model

(1) After the reconnaissance mission begins, each UAV starts from the ship platform at the same time, and the departure time is t = 0.

(2) UAVs that obtain tasks in the initial task assignment are called dispatched UAVs. After the dynamic event occurs, all dispatched UAVs participate in the task reassignment.

(3) Each task is completed by a single UAV.

(4) Every UAV can obtain its own location in real time.

#### 2.2.2. UAV Model

The UAVs are initially carried by the ship platforms. The number of ship platforms is $n_{s}$, the ship platform set is $S=\left\{S_{0},S_{1},\ldots ,S_{n_{s}-1}\right\}$, and the coordinate of $S_{i}$ is $\left[X_{S_{i}},Y_{S_{i}}\right]$. The types and numbers of UAVs carried by each platform are known. The number of UAVs is $n_{u}$, the UAV set is $U=\left\{U_{0},U_{1},\ldots ,U_{n_{u}-1}\right\}$, the cruising speed of $U_{i}$ is $v_{i}$, the maximum range of $U_{i}$ is $mr_{i}$, and the initial coordinate is the coordinate of the ship platform. $Plan=\left\{P_{0},P_{1},\ldots ,P_{n_{u}-1}\right\}$ is the task scheme of UAV formation, which will be updated during task execution. The number of dispatched UAVs is $n_{d},U_{dispatch}=\left\{U_{d}^{1},U_{d}^{2},\ldots ,U_{d}^{n_{d}}\right\}$. Once $U_{d}^{i}$ is damaged, remove $U_{d}^{i}$ from $U_{dispatch}$. When dynamic events occur, the coordinate of $U_{i}$ is $\left[X_{U_{i}},Y_{U_{i}}\right]$; the leftover maximum range of $U_{i}$ is $mrl_{i}$; the list of finished tasks of $U_{i}$ is $TF_{i}=\left\{TF_{i}^{1},TF_{i}^{2},\ldots ,TF_{i}^{n_{f}^{i}}\;\right\}$; the list of leftover tasks of $U_{i}$ is $TL_{i}=\left\{TL_{i}^{1},TL_{i}^{2},\ldots ,TL_{i}^{n_{l}^{i}}\;\right\}$; and $n_{f}^{i}$, $n_{l}^{i}$ are the number of finished tasks and leftover tasks of $U_{i}$, respectively.

#### 2.2.3. Target Model

The initial number of targets is $n_{t}$, the target set is $T=\left\{T_{0},T_{1},\ldots ,T_{n_{t}-1}\right\}$, and the approximate coordinate of $T_{j}$ is $\left[X_{T_{j}},Y_{T_{j}}\right]$. The reconnaissance time window for $T_{j}$ with time window constraint is ${\mathrm{TW}}_{j}=\left[tws_{j},\;twe_{j}\right]$. The radius of the circular reconnaissance route for UAV to reconnaissance target $T_{j}$ is $ar_{j}$. If $n_{new}$ new targets appear, the new target set is $T_{new}=\left\{T_{n}^{1},T_{n}^{2},\ldots ,T_{n}^{n_{new}}\right\}$, the approximate coordinate of $T_{n}^{i}$ is $\left[X_{T_{n}^{i}},Y_{T_{n}^{i}}\right]$, and the radius of circular reconnaissance route is $ar_{T_{n}^{i}}$. If $T_{n}^{i}$ contains a time window constraint, the time window is ${\mathrm{TW}}_{T_{n}^{i}}=\left[tws_{T_{n}^{i}},\;twe_{T_{n}^{i}}\right]$. Meanwhile, these new targets need to be added to the original target set T, and the updated target set is $T=\left\{T_{0},T_{1},\ldots ,T_{n_{t}-1},T_{n_{t}},\ldots ,\;T_{n_{t}+n_{new}-1}\right\}$. If the location of target $T_{j}$ is changed, its coordinates need to be changed to the new location $\left[X_{T_{j}}^{n},Y_{T_{j}}^{n}\right]$. If the time window of target $T_{j}$ is changed, its time window needs to be changed to the new time window $\left[tws_{j}^{n},\;twe_{j}^{n}\right]$.

#### 2.2.4. Decision Variable

Referring to our previous work [22], define $re_{ij}$ as the decision variable, where $i=0,1,\ldots ,n_{u}-1$ and $j=0,1,\ldots ,n_{t}-1$. The value of $re_{ij}$ is 0 or 1; $re_{ij}=1$ indicates that $U_{i}$ conducts reconnaissance on target $T_{j}$ and vice versa.

#### 2.2.5. Constraint Condition

In engineering fields, constraints occur naturally, such as resource limitations, which the agents are subject to [35]. The purpose of task reassignment is to reasonably assign tasks that have not been finished when a dynamic event occurs under the premise of satisfying various constraints and to obtain a new mission plan, denoted as $Plan_{new}=\left\{P_{n}^{0},P_{n}^{1},\ldots ,P_{n}^{n_{new}-1}\right\}$.

(1) Completeness constraint

To ensure the effective completion of the mission and avoid resource waste, each target must be reconnoitered by one UAV:

(1) $$\sum_{i=1}^{n_{u}}re_{ij}=1,\;\forall j=0,1,2,\ldots ,n_{t}-1$$

(2) Leftover maximum range constraint

After $U_{i}$ receives the new mission plan $P_{n}^{i}$, it starts from the current location $\left[X_{U_{i}},Y_{U_{i}}\right]$ and flies to each target point in turn to perform reconnaissance. The remaining flight distance of $U_{i}$ is recorded as $fd_{i}$; suppose $P_{n}^{i}=\left\{T_{i}^{1},T_{i}^{2},\ldots ,T_{i}^{n_{i}}\right\},fd_{i}$ is expressed as Equation (2):

(2) $$fd_{i}=L_{U_{i},\;T_{i}^{1}}+\sum_{k=1}^{n_{i}}2\pi ar_{T_{i}^{k}}+\sum_{k=1}^{n_{i}-1}L_{T_{i}^{k},\;T_{i}^{k+1}}+L_{T_{i}^{n_{i}},S_{U}^{i}}\;$$

where $L_{U_{i},\;T_{i}^{1}}$ is the distance from the current location of $U_{i}$ to target $\;T_{i}^{1}$, $\sum_{k=1}^{n_{i}}2\pi ar_{T_{i}^{k}}$ represents the sum length of the circular reconnaissance route of targets, $\sum_{k=1}^{n_{i}-1}L_{T_{i}^{k},\;T_{i}^{k+1}}$ represents the sum distances of transferring between targets, and $L_{T_{i}^{n_{i}},S_{U}^{i}}$ represents the flight distance of the UAV from the last target to its ship platform.

The leftover maximum range needs to be updated in real time. According to the discussion in Section 2.2.2, the list of finished tasks of $U_{i}$ is $TF_{i}=\left\{TF_{i}^{1},TF_{i}^{2},\ldots ,TF_{i}^{n_{f}^{i}}\;\right\}$, and the $mrl_{i}$ of $U_{i}$ is expressed as Equation (3):

(3) $$mrl_{i}=mr_{i}-\left(L_{S_{U}^{i},TF_{i}^{1}}+\sum_{k=1}^{n_{f}^{i}}2\pi ar_{TF_{i}^{k}}+\sum_{k=1}^{n_{f}^{i}-1}L_{TF_{i}^{k},\;TF_{i}^{k+1}}+L_{TF_{i}^{n_{f}^{i}},U_{i}}\right)$$

where $L_{S_{U}^{i},TF_{i}^{1}}$ represents the distance from the platform to the first finished target, and $L_{TF_{i}^{n_{f}^{i}},U_{i}}$ represents the distance from the last finished target to the current location of $U_{i}$. If $U_{i}$ is exactly at the location of $TF_{i}^{n_{f}^{i}}$, then $L_{TF_{i}^{n_{f}^{i}},U_{i}}=0$.

In order to ensure that $U_{i}$ can successfully complete the mission, it is required that the remaining flight distance of $U_{i}$ cannot be greater than its remaining maximum range:

(4) $$fd_{i}\leq mrl_{i}$$

(3) Time window constraint

Suppose that the time when a dynamic event happens is $t_{d}$. According to $Plan_{new}$, each UAV can obtain the time period for reconnaissance of its targets. Take $U_{i}$ as an example, supposing $P_{n}^{i}=\left\{T_{i}^{1},T_{i}^{2},\ldots ,T_{i}^{n_{i}}\right\}$. The start time of reconnaissance for $T_{i}^{j}$ is $ts_{i}^{j}$, and the end time of reconnaissance for $T_{i}^{j}$ is $te_{i}^{j}$. Then, $ts_{i}^{j}$ and $te_{i}^{j}$ are expressed as Equations (5) and (6):

(5) $$ts_{i}^{j}=\frac{L_{U_{i},\;T_{i}^{1}}+\sum_{k=1}^{j-1}2\pi ar_{T_{i}^{k}}+\sum_{k=1}^{j-1}L_{T_{i}^{k},\;T_{i}^{k+1}}}{v_{i}}+t_{d}$$

(6) $$te_{i}^{j}=ts_{i}^{j}+2\pi ar_{T_{i}^{j}}/v_{i}$$

If $T_{j}$ contains a time window constraint, $ts_{i}^{j}$ and $te_{i}^{j}$ are required to satisfy the following constraints:

(7) $$\{\begin{array}{c} ts_{i}^{j}\geq tws_{j}\\ te_{i}^{j}\leq twe_{j} \end{array}$$

#### 2.2.6. Cost Function

(1) Cost of multi-UAV formation

In order to intuitively evaluate the pros and cons of the final mission plan, we establish the cost function of UAV formation by referring to the previous literatures [22,36].

(8) $$fc=\alpha \cdot CT+\beta \cdot AT+\gamma \times 10^{4}$$

where *CT* represents the mission completion time, which is the moment that the UAV complete the last task; *AT* represents the average flight time—that is, the average time spent by each UAV from departure to return to the platform (except damaged UAVs); and α,β∈[0,1] are proportional coefficients satisfying α+β=1, based on the discussions in literatures [12,37], which are α=β=0.5 here. Plans that do not satisfy the constraints cannot be used as the final mission plan, considering that a good mission plan should enable UAV formation to complete the task with less consumption. Referring to [36], $\gamma \times 10^{4}$ is used to penalize the plans that do not satisfy the constraints in Section 2.2.5. γ is the constraint factor, and the value of γ is 0 or 1: if the mission plan does not meet the constraints, γ=1, so that fc increases by $10^{4}$; otherwise, γ = 0.

(2) Cost of single UAV

Due to the distributed architecture used in online task reassignment, each UAV cannot obtain the overall task mission plan. When updating its mission scheme, a single UAV cannot measure the merits of its scheme through the cost of UAV formation. Therefore, this paper introduces the single cost function to measure the cost of a single UAV. The cost of $U_{i}$ depends on its task scheme, denoted as $c_{i}$, which is calculated by Equation (9):

(9) $$c_{i}=\omega_{1}\cdot ct_{i}+\omega_{2}\cdot rt_{i}+\gamma_{i}\times 10^{4}$$

where $ct_{i}$ represents the time from now to $U_{i}$ completing all tasks according to its scheme $P_{i}$; $rt_{i}$ represents the time from now to $U_{i}$ returning back to platform; $\omega_{1},\omega_{2}$ are proportional coefficients, $\omega_{1}=\omega_{2}=0.5$ here.$\;\gamma_{i}$ is the constraint factor: if $P_{i}$ is satisfied with the constraints, $\gamma_{i}=0$; otherwise, $\gamma_{i}=1$.

## 3. Two-Stage Distributed Task Assignment Algorithm Based on CNP

The advantages of the TS-DTA algorithm mainly include the following three aspects. The first is to achieve the assignment of multiple targets. The two-stage task assignment strategy can avoid the conflicts of multi-targets in the task allocation process, ensuring smooth task allocation. Secondly, tasks can be assigned efficiently. The algorithm is designed based on CNP. The original CNP is improved such that the TS-DTA algorithm can achieve rapid reassignment and ensure the quality of the solution to a certain extent. Thirdly, the communication burden of UAV formation is relatively small. By introducing the bidding benchmark and the bidding strategy based on route distance, the communication times of UAV formation during task assignment are reduced.

### 3.1. Tasks to Be Assigned

Task reassignment is carried out when some tasks cannot be completed due to dynamic events. When executing task reassignment, partial reassignment can be carried out on the basis of the previous mission plan, which can save computing resources and improve the speed of calculation [17,33]. Therefore, the TS-DTA algorithm proposed in this paper adopts the partial reassignment strategy—that is, only part of the targets that need to be reassigned are assigned and these targets are reasonably added to the existing task scheme of dispatched UAVs $U_{dispatch}$.

According to the description in Section 2.1, the targets directly related to dynamic events need to be reassigned, which should be added to the list to be assigned $TL_{un}$ and removed from the current scheme of their corresponding UAVs. Removing some targets may lead to the reconnaissance time of subsequent targets violating the time window constraints, which will interfere with task reassignment. Therefore, it is necessary to remove the subsequent targets that do not meet the constraints from the current task scheme and add them to $TL_{un}$. These targets are called the additional targets of task reassignment. For example, the current task scheme of $U_{i}$ is $TL_{i}=\left\{TL_{i}^{1},TL_{i}^{2},\ldots ,TL_{i}^{n_{l}^{i}}\;\right\}$. When $TL_{i}^{2}$ needs to be reassigned, we remove $TL_{i}^{2}$ from $TL_{i}$; so, $TL_{i}=\left\{TL_{i}^{1},TL_{i}^{3},\ldots ,TL_{i}^{n_{l}^{i}}\;\right\}$, supposing that $TL_{i}^{4}$, $TL_{i}^{6}$ contain time window constraints. According to the new task scheme, the reconnaissance time of $TL_{i}^{4},TL_{i}^{6}$ will be earlier than the beginning of the time window. Then, it is necessary to remove $TL_{i}^{4},TL_{i}^{6}$ from $TL_{i}$ to $TL_{un}$.

Based on the above discussion, the sources of $TL_{un}$ include the leftover targets of damaged UAVs, new targets, the targets whose location changed, the targets whose time window changed, and additional targets. After a dynamic event occurs, the above targets need to be added to $TL_{un}$. The additional targets are confirmed by UAV formation during the task reassignment process.

### 3.2. The Manager UAV and Contractor UAV

When the contract net protocol is used for task assignment, UAVs obtain a conflict-free solution by negotiating with each other. During this period, a manager UAV $U_{m}$ needs to be specified as the manager in the negotiation process. The other UAVs of $U_{dispatch}$ are contractor UAVs, which are denoted as CU. CU bids for tasks issued by $U_{m}$ during the assignment process. In addition, in the algorithm proposed in this paper, the manager UAV $U_{m}$ itself also participates in the task bidding.

From the perspective of balancing the communication distance between UAVs, this paper takes the UAV closest to the center of dispatched UAV formation as the manager and the remaining UAVs as the contractor. During the mission, $U_{m}$ obtains the location and task execution process of each UAV in real time by communicating with CU and switches the manager’s identity in time according to the relative location between UAVs so as to dynamically update $U_{m}$ and CU.

### 3.3. The Framework of TS-DTA

Based on the previous discussion, some of the targets in $TL_{un}$ may contain time window constraints. If the targets with time window constraints are assigned first, the previously assigned targets may violate time window constraints when other targets are inserted later. These targets need to be assigned again, thus affecting the smooth progress of task assignment. Therefore, this paper adopts the two-stage assignment strategy. Targets without time window constraints are assigned in the first assignment stage. On this basis, the second assignment stage is carried out to assign targets with time window constraints.

**Figure 1:**
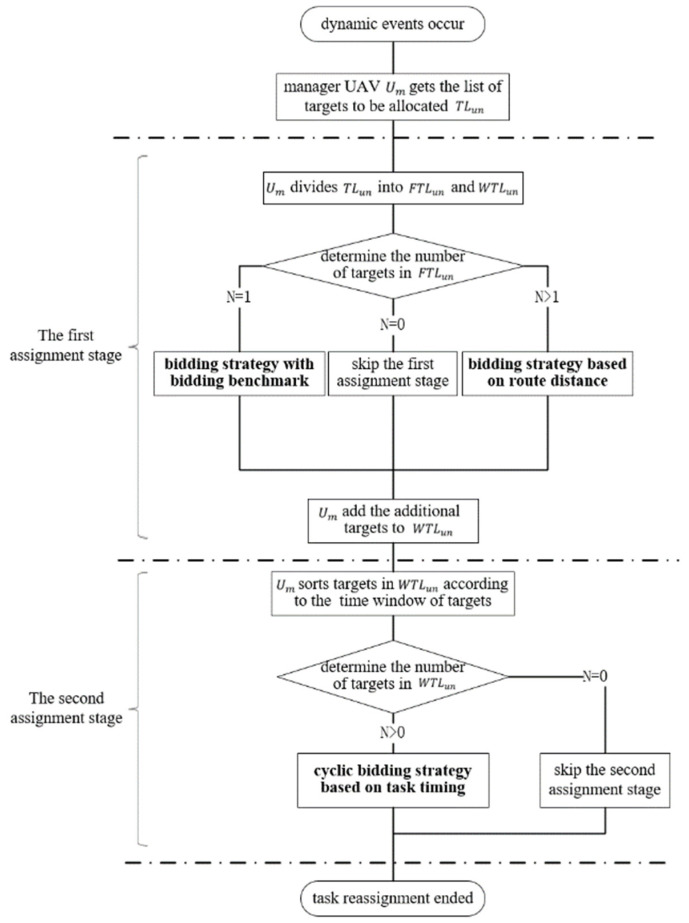
The framework of TS-DTA.

After manager UAV $U_{m}$ obtains the list of tasks to be assigned $TL_{un}$, it is first divided into two sub-lists according to whether the target contains a time window constraint: the task list without time window constraints is $FTL_{un}$; the task list with time window constraints is $WTL_{un}$. At the same time, $U_{m}$ removes every target in $TL_{un}$ from the existing scheme $P_{i}$ of the corresponding UAV $U_{i}$ to obtain the current mission plan, $Plan=\left\{P_{1},P_{2},\ldots ,P_{nu}\right\}$. In particular, for a damaged UAV $U_{b}$, its task list $P_{b}$ needs to be emptied. The specific task assignment architecture is shown in Figure 1.

The objective of the first assignment stage is to assign the targets in $FTL_{un}$ to $U_{dispatch}$. $U_{m}$ needs to determine the number of targets in $FTL_{un}$ first. It needs to be disposed in two cases: If there is only one target $T_{x}$ in $FTL_{un}$, the bidding strategy with bidding benchmark is used for task assignment, as detailed in Section 3.4.2. If there are n (n≥2) targets in $FTL_{un}$, the bidding strategy based on route distance is used for task assignment, as detailed in Section 3.4.3. In addition, $U_{m}$ needs to add additional targets to $WTL_{un}$ in the first assignment stage; the specific process is described in Section 3.4.2 and Section 3.4.3.

The objective of the second assignment stage is to assign the targets in $WTL_{un}$ to $U_{dispatch}$. In the second stage, the cyclic bidding strategy based on task timing is used for task assignment, as detailed in Section 3.5.

### 3.4. The First Assignment Stage

#### 3.4.1. Single-Target Insertion Method

Before we begin to introduce the strategies in the first assignment stage, we need to introduce the single-target insertion method. This method is designed to obtain the optimal new scheme $BP_{i}^{j}$ and the minimum cost increment $mdc_{i}^{j}$ for $U_{i}$ to insert $T_{j}$ into the current scheme $P_{i}$. There may be several targets in $P_{i}$ already; thus, there are different positions of inserting $T_{j}$ into $P_{i}$. The method is to traverse each insertable position of $P_{i}$ and measure the quality of insertion scheme by comparing the cost of $U_{i}$. After the new target is inserted into the current scheme of $U_{i}$, we can obtain a new task scheme of $U_{i}$. The pseudo-code of this method is shown in Algorithm 1.
**Algorithm 1** Single-target Insertion Method$\mathrm{Input}:\;P_{i}$$,\;T_{j}$$\mathrm{Output}:\;BP_{i}^{j}$$,\;mdc_{i}^{j}$1**Let** 
$n=len\left(P_{i}\right)$
$,\;mdc_{i}^{j}$
$=10^{4}$
2$U_{i}$$\;\mathrm{calculates}\;\mathrm{the}\;\cos t\;c_{i}$$\;\mathrm{according}\;\mathrm{to}\;P_{i}$ by Equation (9)3**For** k **in range** (0, n) **do**4
  $CoP_{i}=copy\;\left(P_{i}\right)$
5
  $U_{i}$
$\;\mathrm{insert}\;T_{j}$
$\;\mathrm{into}\;CoP_{i}$
 at the position k
6
  $U_{i}$
$\;\mathrm{calculates}\;\mathrm{the}\;\mathrm{new}\;\cos t\;\mathrm{according}\;\mathrm{to}\;CoP_{i}$
$\;\mathrm{by}\;\mathrm{Equation}\;(9),\;\mathrm{denoted}\;\mathrm{as}\;nc_{i}$
7
  $\mathrm{dc}=nc_{i}$
$-c_{i}$
8
  **If** $dc<mdc_{i}^{j}$
9
    $BP_{i}^{j}$
=
$CoP_{i}$
10
    $mdc_{i}^{j}=dc$
11
  **End if**
12**End for**

Based on the above discussion, before $T_{j}$ is inserted into $P_{i}$, $P_{i}$ satisfies the constraint conditions. If $mdc_{i}^{j}\geq 10^{4}$, it means that the task allocation scheme does not satisfy the constraints after target $T_{j}$ is inserted into $P_{i}$.

#### 3.4.2. Bidding Strategy with Bidding Benchmark

The contract net protocol enables UAVs to negotiate with each other by simulating the bidding process of the market mechanism. One round of bidding includes four stages: initialization, task bidding, winning bids, and signing contracts [38]. The manager and each contractor need to communicate three times in every round of bidding. In this way, with the increase in the number of UAVs, the communication burden of $U_{m}$ is heavier, which reduces the stability of the UAV system. Therefore, this paper proposes a bidding strategy with a bidding benchmark, which requires $U_{m}$ to publish the bidding benchmark at the same time when publishing bidding information. Before bidding, every $U_{i}$ in CU compares its bid with the bidding benchmark. If its bid does not meet the bidding benchmark, $U_{i}$ directly abandons the bidding. In this strategy, the number of communications between UAVs can be reduced. The specific steps are as follows:

$\left(1\right)\;U_{m}$ verifies whether $P_{m}$ meet constraints or not. If $P_{m}$ does not meet constraints, $U_{m}$ removes the targets with time window from $P_{m}$ into $WTL_{un}$. Then, $U_{m}$ finds the best scheme $BP_{m}$ and the minimum cost increment $mdc_{m}$ by inserting $T_{x}$ into $P_{m}$ by single-target insertion method, and the $mdc_{m}$ acts as bidding benchmark. At last, $U_{m}$ sends $T_{x}$, $mdc_{m}$, and Plan to every UAV of CU.

(2) After $U_{i}$($U_{i}\in CU$) receives the bidding information sent by $U_{m},U_{i}$ first verifies whether $P_{i}$ meets the constraints or not. If $P_{i}$ does not meet the constraints, $U_{i}$ removes the targets with time window from $P_{i}$ into $WT_{i}$, which will be sent to $U_{m}$ later. Then, $U_{i}$ find the best scheme $BP_{i}$ and the minimum cost increment $mdc_{i}$ of inserting $T_{x}$ into $P_{i}$ by single-target insertion method. At last, $U_{i}$ compares $mdc_{i}$ with $mdc_{m}$. If $mdc_{i}$ is smaller than $mdc_{m}$, and $mdc_{i}$ is smaller than $10^{4}$, then $U_{i}$ sends $BP_{i}$, $mdc_{i}$, and $WT_{i}$ to $U_{m}$; otherwise, $U_{i}$ sends $WT_{i}$ to $U_{m}$ and gives up the bidding.

(3) After $U_{m}$ receives the bidding information sent by $CU,U_{m}$ puts the additional targets in all received $WT_{i}$ into $WTL_{un}$. If all $U_{i}$ in CU give up the bidding and $mdc_{m}$ is smaller than $10^{4}$, $T_{x}$ is assigned to $U_{m}$. If all $U_{i}$ in CU give up the bidding and $mdc_{m}$ is bigger than $10^{4}$, $T_{x}$ fails to be assigned. Otherwise, $U_{m}$ compares all received mdc and finds the $U_{best}$ with the min mdc, assigns $T_{x}$ to $U_{best}$, updates the task scheme of $U_{best}$ ($P_{best}=BP_{best}$), sends the losing signal to those $U_{i}$ that participated in the bidding (except $U_{best}$), and sends the winning signal to $U_{best}$.

According to the bidding strategy with bidding benchmarks, when the contractor UAV in CU finds that its bid exceeds the bidding benchmark, it directly abandons the bidding, and the $U_{m}$ no longer needs to transmit the information of bidding failure to it. The pseudo-code is shown in Algorithm 2.
**Algorithm 2** Bidding Strategy with Bidding Benchmark$\mathrm{Input}:\;T_{x}$$,\;WTL_{un}$, Plan$,\;U_{m}$, CUOutput: updated Plan$,\;WTL_{un}$1**If** $P_{m}$ does not meet the constraints in Section 2.2.52
  $U_{m}$
$\;\mathrm{removes}\;\mathrm{the}\;\mathrm{targets}\;\mathrm{with}\;\mathrm{time}\;\mathrm{window}\;\mathrm{from}\;P_{m}$
$\;\mathrm{into}\;WTL_{un}$
3**End if**4$U_{m}$$\;\mathrm{get}\;BP_{m}$$,\;mdc_{m}$$\;\mathrm{of}\;\mathrm{inserting}\;T_{x}$$\;\mathrm{into}\;P_{m}$ by *single-target insertion method*5$U_{m}$$\;\mathrm{sends}\;T_{x}$$,\;mdc_{m}$, Plan to every UAV of CU6**For** $U_{i}$ in CU **do (Parallel)**7$\;\mathrm{If}\;P_{i}$ does not meet the constraints in Section 2.2.58$U_{i}$$\;\mathrm{removes}\;\mathrm{the}\;\mathrm{targets}\;\mathrm{with}\;\mathrm{time}\;\mathrm{window}\;\mathrm{from}\;P_{i}$$\;\mathrm{into}\;WT_{i}$9
    **End if**
10  $U_{i}$$\;\mathrm{get}\;BP_{i}$$,\;mdc_{i}$$\;\mathrm{of}\;\mathrm{inserting}\;T_{x}$$\;\mathrm{into}\;P_{i}$ by *single-target insertion method*11$\;\mathrm{If}\;mdc_{i}<mdc_{m}$$\;\mathrm{and}\;mdc_{i}<10^{4}$12
    $U_{i}$
$\;\mathrm{sends}\;BP_{i}$
$,\;mdc_{i}$
$,\;WT_{i}$
$\;\mathrm{to}\;U_{m}$
13
  **Else**
14    $U_{i}$$\;\mathrm{sends}\;WT_{i}$$\;\mathrm{to}\;U_{m}$ and give up the bidding15
  **End if**
16**End for**17$U_{m}$$\;\mathrm{puts}\;\mathrm{the}\;\mathrm{targets}\;\mathrm{in}\;\mathrm{all}\;\mathrm{received}\;WT_{i}$$\;\mathrm{into}\;WTL_{un}$18**If**$\;\mathrm{all}\;U_{i}$ in CU give up the bidding19
  $\mathrm{If}\;mdc_{m}<10^{4}$
20
    $P_{m}=BP_{m}$
21
  **Else**
22    $\;\;\;\;T_{x}$ failed to be assigned23
  **End if**
24**Else**25$U_{m}$ compares all received mdc$\;\mathrm{and}\;\mathrm{finds}\;\mathrm{the}\;U_{best}$ with the min mdc26
    $P_{best}=BP_{best}$
$,\;U_{m}$
$\;\mathrm{sends}\;\mathrm{losing}\mathrm{signal}\;\mathrm{to}\;\mathrm{those}\;U_{i}$
$\;\mathrm{participated}\;\mathrm{in}\;\mathrm{the}\;\mathrm{bidding}\;(\mathrm{except}\;U_{best}$
$)\;\mathrm{and}\;\mathrm{sends}\;\mathrm{winning}\mathrm{signal}\;\mathrm{to}\;U_{best}$
27
     **End if**


The ‘Parallel’ in the pseudo-code indicates that the calculation of each UAV is concurrent. This is because the UAV formation adopts a distributed architecture; so, calculation can be carried out independently based on the computing resources of each UAV. Therefore, the calculation of each UAV is in no order and they do not interfere with each other.

#### 3.4.3. Bidding Strategy Based on Route Distance

When there are multiple targets to be allocated, there will be repeated communications between $U_{m}$ and CU, if targets are tendered one by one. As the number of targets and UAVs increases, the communication burden will be heavy and the stability will be poor. In order to better complete synchronous assignment of multiple targets, this paper proposes a bidding strategy based on route distance. In this strategy, $U_{m}$ assigns bids according to the relative distance between the remaining waypoints of $U_{dispatch}$ (including $U_{m}$) and the targets to be allocated. The specific steps are as follows:

(1)$\;U_{m}$ sends $FTL_{un}$, Plan to every UAV of CU first, then verifies whether $P_{m}$ meet the constraints or not. If $P_{m}$ does not meet constraints, $U_{m}$ removes the targets with time window from $P_{m}$ into $WTL_{un}$. Finally, the distance from each route point contained in $P_{m}$ to each target point in $FTL_{un}$ is calculated, and the distance matrix $DT_{m}$ is obtained.

(2) After receiving the bidding information sent by $U_{m},\;U_{i}\left(U_{i}\in CU\right)$ first verifies whether $P_{i}$ meets the constraints or not. If $P_{i}$ does not meet constraints, $U_{i}$ removes the targets with time window from $P_{i}$ into $WT_{i}$, which will be sent to $U_{m}$ later. Then, $U_{i}$ calculates the distance of every route point in $P_{i}$ to each target in $FTL_{un}$ and obtains the distance matrix $DT_{i}$. Finally, the $DT_{i}$ and $WT_{i}$ are sent to the manager UAV $U_{m}$.

(3) After $U_{m}$ receives the bidding information sent by CU, $U_{m}$ puts the additional targets in all received $WT_{i}$ into $WTL_{un}$. Then, all the distance matrices (including $DT_{m}$) are processed to obtain the task assignment scheme, $Assign=\left\{A_{1},A_{2},\ldots ,A_{n}\right\}$. Assign is a two-dimensional list, in which the elements in sub-list $A_{j}$ are the serial numbers of UAVs in $U_{dispatch}$. The serial numbers are arranged according to the minimum route distance from each UAV to the target $T_{j}$ in ascending order.

(4) $U_{m}$ assigns targets to UAVs according to Assign. At the kth assignment (first set k = 1), $U_{m}$ directly assigns each target to the kth UAV in the corresponding sub-list of Assign for bidding. Thus, $U_{m}$ obtains the assigned task list $ASTL_{k}=\left\{AL_{k}^{1},AL_{k}^{2},\ldots ,AL_{k}^{n_{d}}\right\}$. Then, $U_{m}$ sends the $AL_{k}^{i}$ to each corresponding UAV $U_{i}$ and processes the task list assigned to itself.

(5) After $U_{i}\left(U_{i}\in U_{dispatch}\right)$ receives $AL_{k}^{i}$, for every task $T_{j}$ in $AL_{k}^{i}$, the single-target insertion method is adopted to find the optimal scheme $BP_{i}^{j}$ and the minimum cost increment $mdc_{i}^{j}$ of inserting $T_{j}$ into $P_{i}$. If $mdc_{i}^{j}$ is smaller than $10^{4}$, update the task scheme of $U_{i}$ ($P_{i}=BP_{i}^{j}$), remove $T_{j}$ from $AL_{k}^{i}$, and try to insert the next target in $AL_{k}^{i}$. Otherwise, try to insert the next target in $AL_{k}^{i}$ directly. After all the targets in $AL_{k}^{i}$ are traversed once, the remaining $AL_{k}^{i}$ is sent back to $U_{m}$.

(6) After obtaining feedback from all $U_{dispatch}$, $U_{m}$ merges the targets in all $AL_{k}^{i}$ into a new $FTL_{un}$. Then, it is judged whether $FTL_{un}$ is an empty set: if it is empty, the assignment of $FTL_{un}$ is completed; if not empty, let k=k+1, and then repeat steps (3)–(5). Continue this process until $FTL_{un}$ is an empty set, meaning the task assignment is successful; otherwise, if $k>n_{d}$ ($n_{d}$ is the number of dispatched UAVs), it means the remaining targets in $FTL_{un}$ failed to be assigned.

According to Formula (9), for targets without a time window constraint, the main factor affecting the assignment result is distance. From the perspective of reducing flight distance of UAVs, the target should be assigned to the UAV with the smallest flight distance added by completing the target. Therefore, the bidding strategy based on route distance is proposed. The manager $U_{m}$ directly assigns UAVs to bid for targets, which ensures the quality of the task scheme while reducing communication burden. The pseudo-code is shown in Algorithm 3.
**Algorithm 3** Bidding Strategy Based on Route Distance$\mathrm{Input}:\;FTL_{un}$$,\;WTL_{un}$, Plan$,\;U_{m}$, CU$,\;U_{dispatch}$$,\;n_{d}$Output: updated Plan$,\;WTL_{un}$1$U_{m}$$\;\mathrm{sends}\;FTL_{un}$, Plan to every UAV of CU2**If** $P_{m}$ does not meet the constraints in Section 2.2.53
  $U_{m}$
$\;\mathrm{removes}\;\mathrm{the}\;\mathrm{targets}\;\mathrm{with}\;\mathrm{time}\;\mathrm{window}\;\mathrm{from}\;P_{m}$
$\;\mathrm{into}\;WTL_{un}$
4**End if**5$U_{m}$$\;\mathrm{calculates}\;\mathrm{the}\;\mathrm{distance}\;\mathrm{of}\;\mathrm{every}\;\mathrm{route}\;\mathrm{point}\;\mathrm{to}\;\mathrm{each}\;\mathrm{target}\;\mathrm{in}\;FTL_{un}$$,\;\mathrm{gets}\;DT_{m}$6**For** $U_{i}$ in CU **do (Parallel)**7$\;\mathrm{If}\;P_{i}$ does not meet the constraints in Section 2.2.58
      $U_{i}$
$\;\mathrm{removes}\;\mathrm{the}\;\mathrm{targets}\;\mathrm{with}\;\mathrm{time}\;\mathrm{window}\;\mathrm{from}\;P_{i}$
$\;\mathrm{into}\;WT_{i}$
9
    **End if**
10
    $U_{i}$
$\;\mathrm{calculates}\;\mathrm{the}\;\mathrm{distance}\;\mathrm{of}\;\mathrm{every}\;\mathrm{route}\;\mathrm{point}\;\mathrm{to}\;\mathrm{each}\;\mathrm{target}\;\mathrm{in}\;FTL_{un}$
$,\;\mathrm{gets}\;DT_{i}$
11
    $U_{i}$
$\;\mathrm{sends}\;DT_{i}$
$,\;WT_{i}$
$\;\mathrm{to}\;U_{m}$
12**End for**13$U_{m}$$\;\mathrm{puts}\;\mathrm{the}\;\mathrm{targets}\;\mathrm{in}\;\mathrm{all}\;\mathrm{received}\;WT_{i}$$\;\mathrm{into}\;WTL_{un}$14$U_{m}$$\;\mathrm{processes}\;\mathrm{all}\;DT_{i}$, gets Assign15k = 116**While** $FTL_{un}\;\neq \;\emptyset$$\;\mathrm{and}\;k\leq n_{d}$ **do**17 For j$\;\mathrm{in}\;\mathrm{range}\;1\;\mathrm{to}\;n_{ftl}$ **do**18$\mathrm{Assign}\;FTL_{un}\left[j\right]$ to Assign[j][k]19**End for**20  $U_{m}$$\;\mathrm{gets}\;\mathrm{the}\;\mathrm{assigned}\;\mathrm{task}\;\mathrm{list}\;ASTL_{k}$$\;\mathrm{and}\;\mathrm{sends}\;AL_{k}^{i}$$\;\mathrm{to}\;U_{i}$$\;(AL_{k}^{m}$$\;\mathrm{handled}\;\mathrm{by}\;U_{m}$)21$\;\mathrm{For}\;U_{i}$$\;\mathrm{in}\;U_{dispatch}$ **do (Parallel)**22**For** $T_{j}$$\;\mathrm{in}\;AL_{k}^{i}$ **do**23  $U_{i}$$\;\mathrm{get}\;BP_{i}^{j}$$,\;mdc_{i}$$\;\mathrm{of}\;\mathrm{inserting}\;T_{j}$$\;\mathrm{into}\;P_{i}$ by *single-target insertion method*24$\;\mathrm{If}\;mdc_{i}<10^{4}$25
     $P_{i}=BP_{i}^{j}$
$,\;\mathrm{delete}\;T_{j}$
$\;\mathrm{from}\;AL_{k}^{i}$
26
  **End if**
27**End for**28$U_{i}$$\;\mathrm{sends}\;AL_{k}^{i}$$\;\mathrm{to}\;U_{m}$29
  **End for**
30
  k=k+1
31
  $U_{m}$
$\;\mathrm{put}\;\mathrm{the}\;\mathrm{targets}\;\mathrm{in}\;\mathrm{all}\;AL_{k}^{i}$
$\;\mathrm{into}\;FTL_{un}$
32**End while**33**If** 
$FTL_{un}=\emptyset$
34  All tasks without time window have been assigned successfully35**Else**36    $\;\mathrm{Tasks}\;\mathrm{in}\;FTL_{un}$ failed to be assigned

### 3.5. The Second Assignment Stage

The second assignment stage mainly relies on the cyclic bidding strategy based on task timing to complete the task assignment since targets in $WTL_{un}$ are constrained by time windows, and the end times of time windows are different. The end time of the time window of $T_{j}$ is denoted as $twe_{j}$. It is a better choice to first assign the targets with an earlier end time of time window. Otherwise, some subsequent targets may need to be inserted ahead of the targets assigned previously. This insertion would change the mission process. As a result, the reconnaissance time of previously assigned tasks may not satisfy its time window constraint. In order to ensure that the task assignment can be carried out more smoothly, the targets in $WTL_{un}$ are firstly sorted according to the end time of time window, and then allocated one by one. At the same time, after the post-order targets are assigned, the pre-order targets that fail to be assigned could be successfully allocated. In order to enable more targets to be successfully assigned, we use a cyclic bidding method. In addition, in order to reduce the communication burden and improve the efficiency of assignment, similar to Section 3.4.1, the cyclic bidding strategy introduces a bidding benchmark when assigning each target in $WTL_{un}$. The specific process is as follows:

(1)$\;U_{m}$ finds the best scheme $BP_{m}^{k}$ and the minimum cost increment $mdc_{m}^{k}$ of inserting the kth (first set k = 1) target in $WTL_{un}$ into $P_{m}$ by single-target insertion method. The $mdc_{m}^{k}$ acts as bidding benchmark. Then, $U_{m}$ sends $T_{w}^{k}$, $mdc_{m}^{k}$, and Plan to every UAV of CU.

(2) After receiving the bidding information sent by $U_{m},\;\;U_{i}\left(U_{i}\in CU\right)$ finds the best scheme $BP_{i}^{k}$ and the minimum cost increment $mdc_{i}^{k}$ of inserting $T_{w}^{k}$ into $P_{m}$ by single-target insertion method. Then, $U_{i}$ compares $mdc_{i}^{k}$ with $mdc_{m}^{k}$. If $mdc_{i}^{k}$ is smaller than $mdc_{m}^{k}$, and $mdc_{i}^{k}$ is smaller than $10^{4}$, then $U_{i}$ sends $BP_{i}^{k}$ and $mdc_{i}^{k}$ to $U_{m}$; otherwise, $U_{i}$ sends a give up signal to $U_{m}$ and gives up the bidding.

(3) After $U_{m}$ receives the bidding information sent by CU, $U_{m}$ compares all received mdc and finds the $U_{best}$ with the min mdc; then, it assigns $T_{w}^{k}$ to $U_{best}$, updates the task scheme of $U_{best}$ ($P_{best}=BP_{best}$), sends losing signal to those $U_{i}$ that participated in the bidding (except $U_{best}$), and sends winning signal to $U_{best}$. Then, $T_{w}^{k}$ is removed from $WTL_{un}$. If all $U_{i}$ in CU give up the bidding and $mdc_{m}^{k}$ is smaller than $10^{4}$, $T_{w}^{k}$ is assigned to $U_{m}$.

(4) Repeat steps (1)–(3) (increase k by 1 each time) until all targets in $WTL_{un}$ complete a traversal.

(5) Determine whether $WTL_{un}$ is an empty set: if yes, the task allocation is complete; otherwise, it is judged whether the target in $WTL_{un}$ is consistent with the target after the last traversal. If not, let k = 1 and repeat steps (1)–(4); if yes, it means that the existing tasks in $WTL_{un}$ can no longer be successfully assigned, the second assignment stage is terminated, and targets in $WTL_{un}$ failed to be assigned.

The pseudo-code of this strategy is shown in Algorithm 4.
**Algorithm 4** Cyclic Bidding Strategy Based on Task Timing$\mathrm{Input}:\;WTL_{un}$, Plan$,\;U_{m}$, CUOutput: updated Plan1$\mathrm{Co}WTL_{un}$$=\mathrm{copy}\;(WTL_{un}$)2**While** $WTL_{un}\neq \;\emptyset$$\;\mathrm{or}\;CoWTL_{un}\neq WTL_{un}$ **do**3
  $\mathrm{Co}WTL_{un}=\mathrm{copy}\;\left(WTL_{un}\right)$
4  **For** k$\;\mathrm{in}\;\mathrm{range}\;(1,\;n_{wtl}$) **do**5    $U_{m}$$\;\mathrm{gets}\;BP_{m}^{k}$$,\;mdc_{m}^{k}$$\;\mathrm{of}\;\mathrm{inserting}\;T_{w}^{k}$$\;\mathrm{into}\;P_{m}$ by *single-target insertion method*6
    $U_{m}$
$\;\mathrm{sends}\;T_{w}^{k}$
$,\;mdc_{m}^{k}$
, Plan
 to every UAV of CU
7    **For** $U_{i}$ in CU **do (Parallel)**8     $U_{i}$$\;\mathrm{gets}\;BP_{i}^{k}$$,\;mdc_{i}^{k}$$\;\mathrm{of}\;\mathrm{inserting}\;T_{w}^{k}$$\;\mathrm{into}\;P_{i}$ by *single-target insertion method*9
     $\;\mathrm{If}\;mdc_{i}^{k}<mdc_{m}^{k}$
$\;\mathrm{and}\;mdc_{i}^{k}<10^{4}$
10
      $U_{i}$
$\;\mathrm{sends}\;BP_{i}^{k}$
$,\;mdc_{i}^{k}\;$
$\mathrm{to}\;U_{m}$
11
     **Else**
12      $U_{i}$ give up the bidding13
     **End if**
14
      **End for**
15      **If**$\;\mathrm{all}\;U_{i}$ in CU give up the bidding 16
    $\;\mathrm{If}\;mdc_{m}<10^{4}$
19      $P_{m}=BP_{m}^{k}$$,\;U_{m}$$\;\mathrm{delete}\;T_{w}^{k}$ from $WTL_{un}$20
     **End if**
21**Else**22
  $U_{m}$
 compares all received mdc
$\;\mathrm{and}\;\mathrm{finds}\;\mathrm{the}\;U_{best}$
 with the min mdc
23
    $P_{best}=BP_{best}$
$,\;U_{m}$
$\;\mathrm{sends}\;\mathrm{losing}\mathrm{signal}\;\mathrm{to}\;\mathrm{those}\;U_{i}$
$\;\mathrm{participated}\;\mathrm{in}\;\mathrm{the}\;\mathrm{bidding}\;(\mathrm{except}\;U_{best}$
$)\;\mathrm{and}\;\mathrm{sends}\;\mathrm{winning}\mathrm{signal}\;\mathrm{to}\;U_{best}$
24
     **End if**
25
      **End for**
26
     **End while**


## 4. Performance Analysis of TS-DTA

In order to comprehensively analyze the performance of the TS-DTA algorithm, a series of experiments are conducted based on the DE-MCRTR model. All algorithms are implemented in Python 3.9, the IDE is PyCharm, the computer CPU is an AMD Ryzen 5-5600H, and the CPU clock speed is 3.30 GHz. The distance unit in the text is kilometer (km) and the time unit is second (s). In order to simplify the expression, the units are omitted in the following text.

### 4.1. Validation of Effectiveness

#### 4.1.1. Background Information

In order to verify the effectiveness of the TS-DTA algorithm in the DE-MCRTR model, we conducted a set of simulation experiments. Referring to existing literature [12,22,37], this article assumes that there are four ship platforms, each equipped with two UAVs. The cruising speeds and maximum ranges of different types of UAVs are different. The ship platforms are distributed in the area of [0, 100]×[0, 100]; we randomly initialize the location of the four platforms. The initialization information and related parameters are shown in Table 1.

The targets are distributed in the area of [100, 300]×[100, 300]. The radius of the circular routes of targets are initialized randomly within [1, 3]. We assume that there are twenty targets, of which five targets contain time window constraints. Similarly, this article randomly initializes the locations of the twenty targets within the given range and randomly selects five targets to set the time window constraints. The initialization information of targets is shown in Table 2.

**Figure 2:**
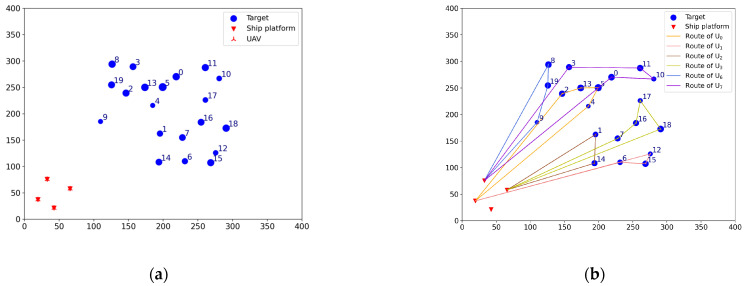
(**a**) Initial situation. (**b**) Initial mission plan.

Before the mission starts, the offline task assignment algorithm is first used to obtain the initial mission plan: Plan = [[2, 13, 5, 4], [6, 15, 12], [1, 14], [7, 16, 17, 18], [], [], [8, 19, 9], [0, 10, 11, 3]]. The Plan is a two-dimensional list, where the sub-list represents the scheme of each UAV. The numbers in the sub-list represent the targets’ number. The same is true later. According to the initial mission plan, a total of six UAVs are dispatched to carry out the reconnaissance mission: $U_{dispatch}=\left[U_{0},U_{1},U_{2},U_{3},U_{6},U_{7}\right]$, $n_{d}=6$. The initial situation and the initial mission plan are shown in Figure 2a,b. The numbers next to the targets icon indicate targets’ number, the same is true later.

#### 4.1.2. Task Reassignment in the Case of UAV Damage

**Figure 3:**
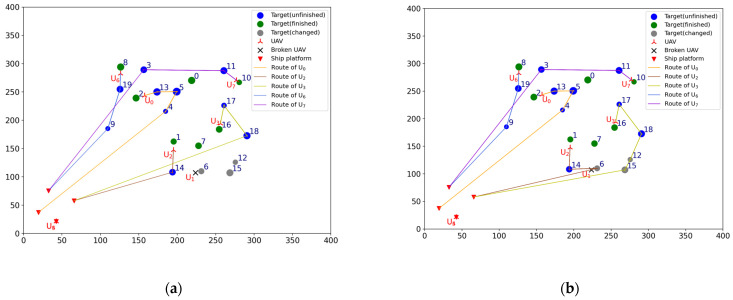
(**a**) The battlefield situation of test 1. (**b**) Result of task reassignment in test 1.

To verify the effectiveness of the TS-DTA algorithm in the case of UAV damage, test 1 is conducted. The moment when the drone departs from the ship platform is t = 0. Assuming t = 1200, $U_{1}$ is attacked by the enemy and loses the ability to execute tasks. In the simulation experiment, the location and task execution status of each UAV is calculated based on the initial mission plan. By this way, the finished task list of UAV formation is TF=[[2], [], [1], [7, 16], [], [], [8], [0, 10]], the leftover task list of UAV formation is TL=[[13, 5, 4], [6, 15, 12], [14], [17, 18], [], [], [19, 9], [11, 3]], and $TL_{1}=\left[6,\;15,\;12\right]$ is the leftover task list of $U_{1}$. The tasks in $TL_{1}$ need to be assigned to $U_{dispatch}$. So, the list of tasks to be assigned is $TL_{un}=\left[6,\;15,\;12\right]$, and $U_{dispatch}=\left[U_{0},U_{2},U_{3},U_{6},U_{7}\right]$. The current battlefield situation is shown in Figure 3a.

In Figure 3, the green circular icons represent the targets that have been reconnoitered, the blue circular icons represent the targets that have not been finished, the gray circular icons represent the targets that have changed, and the solid lines of different colors represent the remaining flight routes of UAVs according to the current mission plan. The UAVs that have not been given a task are still located on ship platforms; so, the icons of these UAVs and ship platforms overlap.

**Figure 4:**
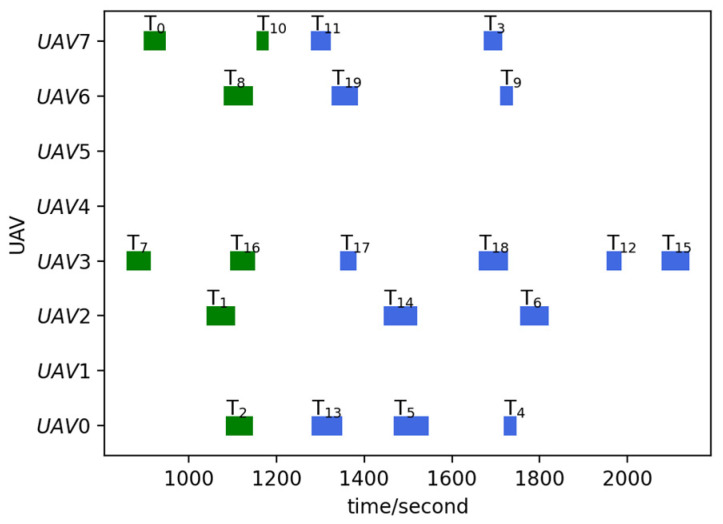
Reconnaissance schedules in test 1.

Run the TS-DTA algorithm to assign tasks in $TL_{un}$. The new mission plan is $Plan_{new}=$ [[13, 5, 4], [], [14, 6], [17, 18, 12, 15], [], [], [19, 9], [11, 3]. The cost of multi-UAV formation is fc=2051.28. So, $T_{6}$ is assigned to $U_{2}$, and $T_{12}$, $T_{15}$ are assigned to $U_{3}$. According to $Plan_{new}$, the remaining routes of UAVs are shown in Figure 3b. The reconnaissance schedules of targets are shown in Figure 4. The green icons represent the reconnaissance time of the targets that have been reconnoitered, and the blue icons represent the reconnaissance time of the targets that will be reconnoitered. The same is true later.

From Figure 3b, we can find that $T_{6}$ will be reconnoitered by $U_{2}$ and $T_{12},T_{15}$ will be reconnoitered by $U_{3}$. Combining Table 2 and Figure 4, $Plan_{new}$ can ensure that all targets with time windows are reconnoitered within the time window.

**Figure 5:**
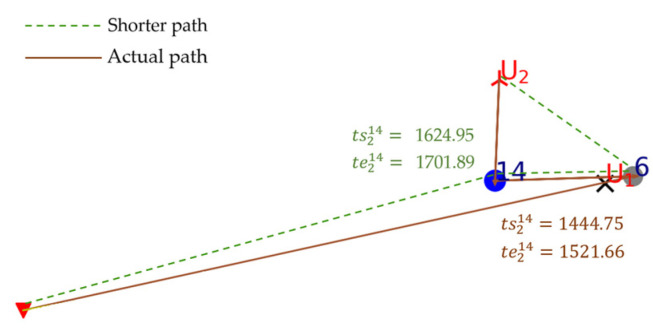
Routes comparison.

According to $Plan_{new}$, $T_{6}$ is reconnoitered after $T_{14}$ in the scheme of $U_{2}$. In Figure 5, there are two possible routes for $U_{2}$ to finish $T_{6}$ and $T_{14}$. One is the shorter route, shown by the green dotted line, where the start and end times of the $U_{2}$ reconnaissance of $T_{14}$ are marked in green font in the figure. Another is the actual route, shown by the brown solid line, where the start and end times of the $U_{2}$ reconnaissance of $T_{14}$ are marked in brown font in the figure. $ts_{i}^{j}$ represents the $U_{i}$ start time to reconnoiter $T_{j}$; $te_{i}^{j}$ represents the $U_{i}$ end time reconnoitering $T_{j}$. From Figure 5, we can find that in the shorter route $T_{6}$ is reconnoitered before $T_{14}$. However, if the shorter route is adopted, $ts_{2}^{14}=1624.95$ and $te_{2}^{14}=1701.89$, which obviously contradicts $TW_{14}=\left[1000,\;1600\right]$. So, the shorter route (green dotted line) is abandoned.

#### 4.1.3. Task Reassignment in the Case of New Target Occurrence

To verify the effectiveness of the TS-DTA algorithm in the case of finding new targets, test 2 is conducted. Suppose that at t = 1300, the early warning aircraft detects three new targets: $T_{n}^{1}$, $T_{n}^{2}$*,* and $T_{n}^{3}$. The location of $T_{n}^{1}$ is $LOC_{T_{n}^{1}}=$ [217.00, 153.00], the radius of the annular route $ar_{T_{n}^{1}}\;$= 1.50, and the reconnaissance time window is $TW_{T_{n}^{1}}=\left[1500,\;1800\right]$. The location of $T_{n}^{2}$ is $LOC_{T_{n}^{2}}=$ [130.00, 206.00], and the radius of the annular route $ar_{T_{n}^{2}}$ = 2.30, without a reconnaissance time window. The location of $T_{n}^{3}$ is $LOC_{T_{n}^{3}}=$ [241.00, 263.00], and the radius of annular route $ar_{T_{n}^{2}}$ = 1.90, without a reconnaissance time window.

**Figure 6:**
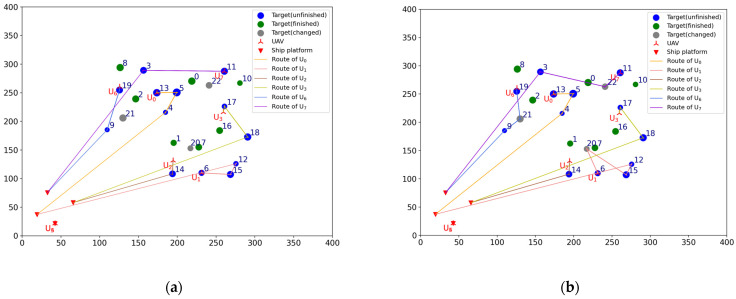
(**a**) The battlefield situation of test 2. (**b**) Result of task reassignment in test 2.

According to Section 2.2.3, new targets need to be added into the original target set T. The original target number is 20, numbered from 0 to 19. So, $T_{n}^{1}$ is numbered 20, denoted as $T_{20}$. Similarly, $T_{n}^{2}$ and $T_{n}^{3}$ are numbered 21 and 22, which are denoted as $T_{21}$ and $T_{22}$, respectively. The current battlefield situation is shown in Figure 6a.

The list of tasks to be allocated is $TL_{un}=\left[20,\;21,\;22\right]$. The UAVs participating in task reassignment are $U_{dispatch}=\left[U_{0},U_{1},U_{2},U_{3},U_{6},U_{7}\right]$. The finished task list of UAV formation is TF=[[2], [], [1], [7, 16], [], [], [8], [0, 10]], and the leftover task list of UAV formation is TL=[[13, 5, 4], [6, 15, 12], [14], [17, 18], [], [], [19, 9], [11, 3]].

**Figure 7:**
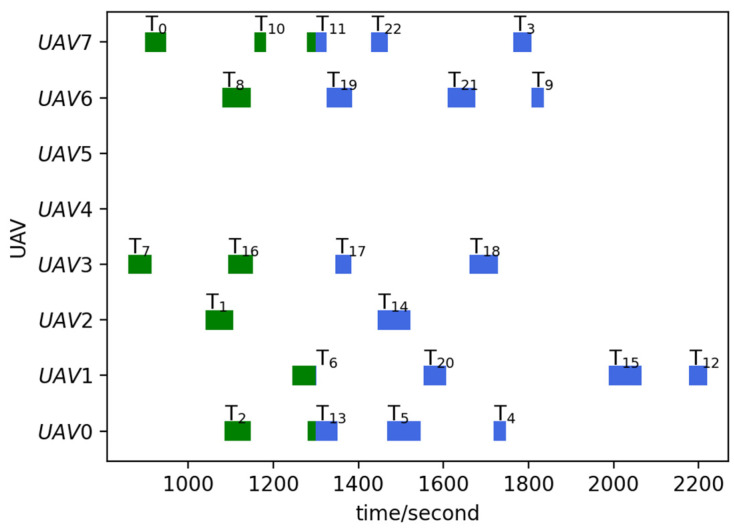
Reconnaissance schedules in test 2.

Run the TS-DTA algorithm to obtain the new task plan: $Plan_{new}=$ [[13, 5, 4], [6, 20, 15, 12], [14], [17, 18], [], [], [19, 21, 9], [11, 22, 3]]. The cost of multi-UAV formation is fc=2166.98. According to $Plan_{new}$, $T_{20}$ is assigned to $U_{1}$, $T_{21}$ is assigned to $U_{6}$, and $T_{22}$ is assigned to drone $U_{7}$. The result is shown in Figure 6b. The reconnaissance schedules of targets are shown in Figure 7. Analysis shows that $T_{21}$, $T_{22}$ are assigned through bidding strategy based on route distance. So, $T_{21}$ and $T_{22}$ are assigned to $U_{6}$ and $U_{7}$, respectively, which are judged from the route distance.

It is reasonable to assign to $U_{1}$ and $U_{2}$ from the perspective of flight distance. However, $T_{20}$ needs to be completed within the time window of [1500, 1800], which conflicts with the time window of $T_{14}$ that belongs to $U_{1}$. So, $T_{20}$ is finally assigned to $U_{2}$.

#### 4.1.4. Task Reassignment in the Case of Changing the Location of Targets

**Figure 8:**
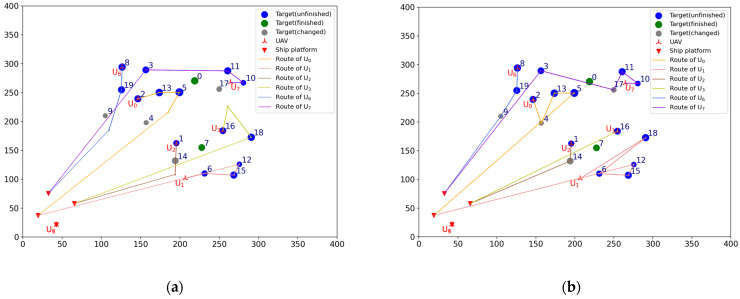
(**a**) The battlefield situation of test 3. (**b**) Result of task reassignment in test 3.

To verify the effectiveness of the TS-DTA algorithm in the case of changing the location of targets, test 3 is conducted. It is assumed that at time t = 1100, the early warning aircraft detects that four targets have moved to new locations as follows: $T_{4}$ moves from [184.89, 215.84] to [157.00, 198.00], $T_{9}$ moves from [109.69, 185.35] to [105.00, 210.00], $T_{14}$ moves from [193.87, 108.35] to [194.00, 132.00], and $T_{17}$ moves from [260.90, 226.18] to [250.00, 256.00]. The list of tasks to be allocated is $TL_{un}=\left[4,\;9,\;14,\;17\right]$. Among them, $T_{4}$ and $T_{14}$ contain time window constraints, and $T_{9}$ and $T_{17}$ do not contain time window constraints. The current battlefield situation is shown in Figure 8a.

The lines in Figure 8a represent the original planned routes of UAVs. Because the locations of $T_{4}$, $T_{9}$, $T_{14}$, and $T_{17}$ are changed, UAVs can no longer finish the reconnaissance of these moved targets according to the original planned route. So, we need to reassign these moved targets and update the flight routes according to $Plan_{new}$ to ensure that all tasks can be completed. Now, the finished task list of UAV formation is TF=[[], [], [], [7], [], [], [], [0]], and the leftover task list of UAV formation is TL= [[2, 13, 5], [6, 15, 12], [1], [16, 18], [], [], [8, 19], [10, 11, 3]]. The UAVs participating in the assignment are $U_{dispatch}=\left[U_{0},U_{1},U_{2},U_{3},U_{6},U_{7}\right]$.

**Figure 9:**
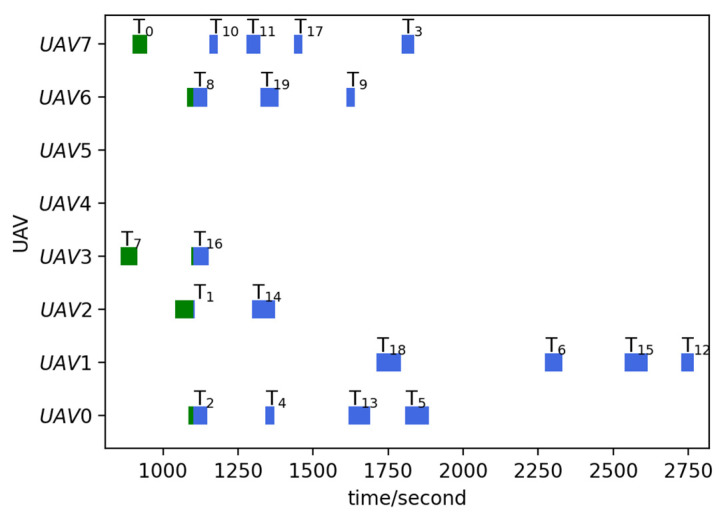
Reconnaissance schedules in test 3.

Run the TS-DTA algorithm to obtain the new task plan: $Plan_{new}=$ [[2, 4, 13, 5], [18, 6, 15, 12], [1, 14], [16], [], [], [8, 19, 9], [10, 11, 17, 3]]; $T_{4},T_{9},T_{14},T_{17}$ are assigned to $U_{0},U_{6},U_{2},U_{7}$, respectively. The cost of multi-UAV formation is fc=2440.83. The remaining routes are shown in Figure 8b, and the reconnaissance schedules of targets are shown in Figure 9. Although $T_{9}$ and $T_{14}$ have moved, the range of motion is not large; thus, they are assigned to the original UAV and the task orders are not changed. As for $T_{4}$, the time window constraint cannot be satisfied due to the change in location. After reassignment, the task execution order of $U_{0}$ is adjusted to ensure that $T_{4}$ is reconnoitered within its time window.

For $U_{3}$, because the location of $T_{17}$ is changed, the algorithm first removes $T_{17}$ from the task list of $U_{3}$, which leads to the advance in the reconnaissance time of $T_{18}$. As a result, the time window of $T_{18}$ cannot be satisfied. So, $T_{18}$ is the additional target, which should be added to $TL_{un}$. Finally, $T_{18}$ is assigned to $U_{0}$, and $T_{17}$ is assigned to $U_{7}$, which is nearer to $T_{17}$. According to $Plan_{new}$, $U_{0}$ first reconnoiters $T_{18}$ and then performs subsequent tasks. By this way, the completion time of subsequent tasks are delayed; however, the time window constraints of all targets are satisfied.

#### 4.1.5. Task Reassignment in the Case of Changing the Time Window of Targets

**Figure 10:**
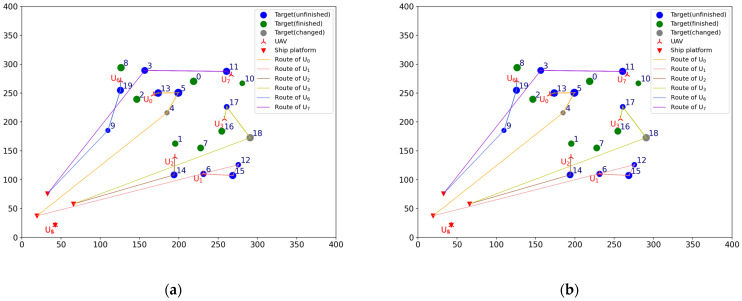
(**a**) The battlefield situation of test 4; (**b**) Result of task reassignment in test 4.

To verify the effectiveness of the TS-DTA algorithm in the case of changing the time window of targets, test 4 is conducted. Assuming that at time t=1250, the time windows of $T_{4}$ and $T_{18}$ are changed. The new time windows are $TW_{4}=\left[1400,\;1700\right]$ and $TW_{18}=\left[1300,\;1600\right]$. The current battlefield situation is shown in Figure 10a.

Now, the finished task list of UAV formation is TF= [[2], [], [1], [7, 16], [], [], [8], [0, 10]] and the leftover task list of UAV formation is TL= [[13, 5], [6, 15, 12], [14], [17], [], [], [19, 9], [11, 3]]. According to the initial mission plan, the start and end times of reconnaissance for $T_{4}$ and $T_{18}$ are [1717.29, 1747.38] and [1661.72, 1728.01], which obviously does not meet the new time window constraints. So, the list of tasks to be allocated is $TL_{un}=\left[4,\;18\right]$, and the UAVs participating in the assignment are $U_{dispatch}=\left[U_{0},U_{1},U_{2},U_{3},U_{6},U_{7}\right]$.

**Figure 11:**
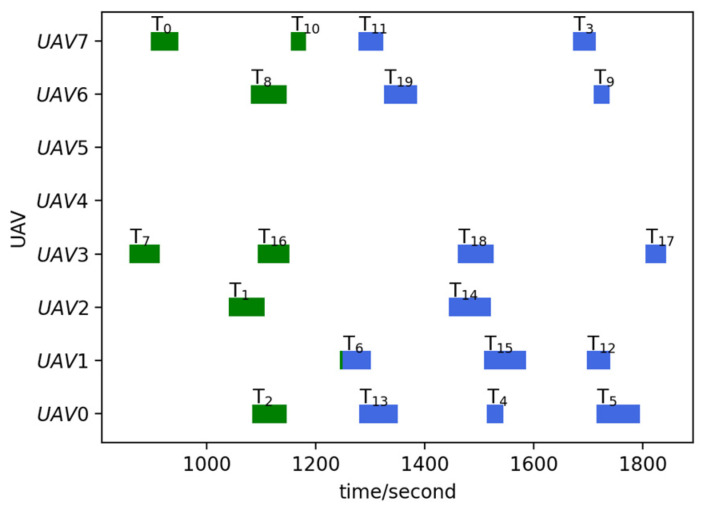
Reconnaissance schedules in test 4.

Run the TS-DTA algorithm to obtain a new mission plan: $Plan_{new}=$ [[13, 4, 5], [6, 15, 12], [14], [18, 17], [], [], [19, 9], [11, 3]]. The cost of multi-UAV formation is fc=1958.39. The results are shown in Figure 10a and Figure 11. According to the initial plan, the task scheme of $U_{0}$ is $P_{0}=\left[2,\;13,\;5,\;4\right]$ and the task scheme of $U_{3}$ is $P_{3}=\left[7,\;16,\;17,\;18\right]$. So, in the initial plan, $T_{4}$ belongs to $U_{0}$ and $T_{18}$ belongs to $U_{3}$. It can be seen from $Plan_{new}$ that both $T_{4}$ and $T_{18}$ are assigned to the UAV they originally belonged to. However, compared with the initial plan, the orders of tasks in $P_{0}$ and $P_{3}$ have changed. According to $Plan_{new}$, $U_{0}$ reconnoiters $T_{4}$ and then reconnoiters $T_{5}$, and $U_{3}$ reconnoiters $T_{18}$ and then reconnoiters $T_{17}$. This is because the time windows of $T_{4}$ and $T_{18}$ have advanced.

### 4.2. Comparative Analysis

#### 4.2.1. Analysis of Communication Simplification Effect

The communication times mean the total times that UAVs communicate with each other during the process of task assignment. This paper reduces the communication times of UAV formation by introducing a bidding benchmark and assignment strategy based on route distance into the TS-DTA algorithm. Gao et al. [34] proposed a CNP-based algorithm to solve the task reassignment problem, in which targets are allocated one by one. In order to analyze the simplification effect of the TS-DTA algorithm on the communication process, we carried out some comparative experiments. Since the CNP-based algorithm [34] is only applicable to the two cases of UAV damage and new target occurrence, we only consider the above two dynamic events when setting up comparative experiments.

In the experiment, we randomly generate some targets that need to be reconnoitered in the range of [100, 300] × [100, 300] as new targets. The specific data are shown in Table 3.

We conducted the following three sets of tests: UAV damage, new target appearance, and new target appearance at the same time as UAV damage. Experiments are based on the initial mission plan described in Section 4.1.1, and different dynamic events are introduced for comparative experiments.

The dynamic events are introduced at time t = 1000 in the first set of experiments, recorded as test 5. The leftover task list of UAV formation is TL= [[2, 13, 5, 4], [6, 15, 12], [1, 14], [16, 17, 18], [], [], [8, 19, 9], [10, 11, 3]]. By changing the serial number and numbers of damaged UAVs, comparative experiments are carried out. The experimental results are shown in Table 4. In the table, $n_{d}$ represents the number of UAVs in $U_{dispatch}$ and $n_{un}$ represents the number of targets to be allocated.

It can be seen from the results that under various experimental conditions, the communication times of the CNP-based algorithm are greater than those of the TS-DTA algorithm. It is noted that the communication times of the TS-DTA algorithm are not stable even if $n_{d}$ and $n_{un}$ are constant, which is mainly caused by three reasons: First, the bidding benchmark is introduced in the algorithm and the number of contractors that meet the bidding benchmark in the bidding process is not certain. Second, the number of assignments in the bidding strategy based on the route distance is not stable. Third, the number of cycles of the cyclic bidding strategy based on task timing is not stable.

The dynamic events are introduced at time t = 1200 in the second set of experiments, recorded as test 6. The leftover task list of UAV formation is TL = [[13, 5, 4], [6, 15, 12], [14], [17, 18], [], [], [19, 9], [11, 3]]. By introducing different numbers of new targets for comparative analysis, the experimental results are shown in Table 5. It can be seen from the results that as the number of new targets increases, the communication times of the CNP-based algorithm increases linearly. However, the communication times of the TS-DTA algorithm are significantly less than those of the CNP-based algorithm, and as the number of targets increases, the advantage is more obvious.

The dynamic events are introduced at time t = 1200 in the third set of experiments, recorded as test 7. A comparative analysis was performed by changing the number of damaged UAVs and the number of new targets. The experimental results are shown in Table 6. It can be seen from the results that the number of communication times of the TS-DTA algorithm is significantly lower than that of the CNP-based algorithm in various cases.

#### 4.2.2. Analysis of Solution Speed and Solution Quality

In order to analyze the solution speed and solution quality of the TS-DTA algorithm, we conducted a set of comparative experiments. In addition to the CNP-based algorithm mentioned in Section 4.2.1, we add two centralized task assignment algorithms for comparison, including RPSO [39] and IEPPSO [22]. The solution speed and solution quality of RPSO and IEPPSO are related to the population size of particles. In order to analyze the performance of the algorithm more comprehensively, the task assignment effects of RPSO and IEPPSO algorithms with population sizes of 100, 300, and 500 are compared in each experiment. In the experiment, the number of iterations of the centralized algorithm is set to 200.

In the experiment, the number of targets to be reassigned is set to 2, 4, 6, 8, and 10, respectively. The performance is analyzed by comparing the CPU running time ($t_{cpu}$) and the overall cost of UAV formation (fc) in various cases. Due to the randomness of the results of RPSO and IEPPSO algorithms, in order to ensure the objectivity of experiments, the RPSO and IEPPSO algorithms are run 20 times in each test, and the average of the 20 results are taken as the final result. The results are shown in Table 7.

**Figure 12:**
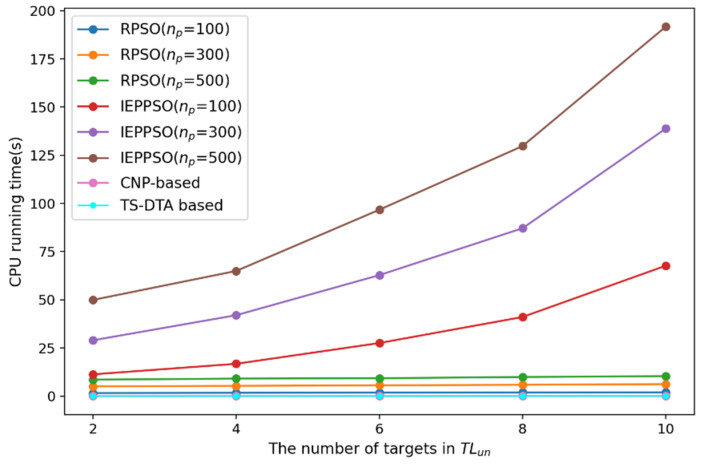
CPU running time.

**Figure 13:**
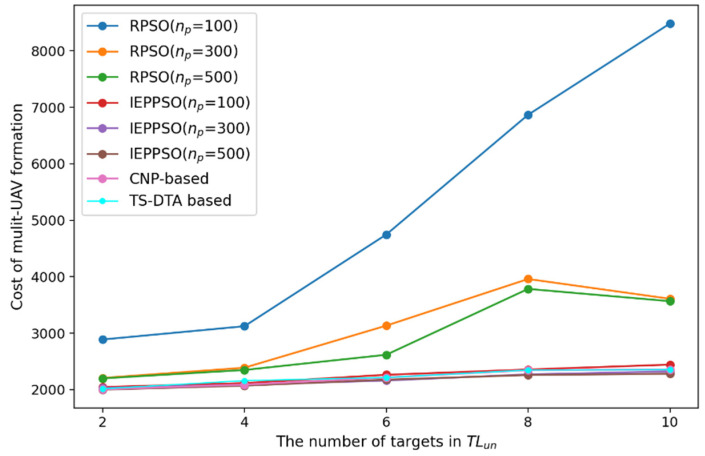
Cost of UAV formation.

According to the results of Table 7, the paper draws a line chart of CPU running time changing with the number of targets to be allocated, as shown in Figure 12, and draws a line chart of overall cost changing with the number of targets to be allocated, as shown in Figure 13.

From Figure 12, we can intuitively see that the CPU running time of the TS-DTA algorithm and the CNP-based algorithm is the shortest, followed by the RPSO algorithm, and that of the IEPPSO algorithm is the longest. As the population size increases, the time consumption of the RPSO and IEPPSO algorithms increase. Based on the experimental results in Section 4.2.1, the CNP-based algorithm requires a large amount of communication for task assignment. In the experiment, we did not consider the time cost of communication, and a large amount of communication will lead to a slower solution speed in actual task assignment.

From Figure 13, we can see that as the number of targets increases, the solving ability of the RPSO algorithm becomes weaker and the proportion of solutions that do not meet the constraints becomes higher, resulting in an overall higher cost value. The solving ability of the IEPPSO algorithm is relatively stable, and when the population size is 500, the solution of IEPPSO algorithm is optimal in various situations. However, combined with Figure 12, we find that the CPU running time of the IEPPSO algorithm significantly increases with the increase in the number of targets, which reflects that the IEPPSO algorithm improves the quality of the solution by increasing the computational load. So, the IEPPSO algorithm is not applicable in dynamic environments with high requirements for solving speed. Besides, the quality of solutions of the TS-DTA algorithm and the CNP-based algorithm are similar, and there is not much difference compared with the IEPPSO algorithm with a population size of 500.

Finally, considering the solving speed, communication burden, and solution quality, the TS-DTA algorithm is more suitable for solving the task reassignment problem in dynamic environments.

## 5. Conclusions and Future Work

In this paper, a model of multi-UAV cooperative reconnaissance task reassignment is established to comprehensively consider many dynamic events including UAV damage, new target occurrence, location change of the target, and time window change of the target. Then, a two-stage distributed task assignment algorithm (TS-DTA) based on the improved contract net protocol is presented to realize the rapid reassignment of multiple targets, reduce the communication burden of multi-UAV formation, and ensure the quality of task assignment to a certain extent. Finally, the experimental results show that the proposed TS-DTA algorithm can efficiently solve the task reassignment problem in dynamic environments while effectively reducing the communication burden of UAV formation.

Our future works will focus mainly on the two following aspects. Firstly, the TS-DTA algorithm will be further optimized to improve efficiency. Secondly, the task assignment and reassignment of reconnaissance UAVs and attack UAVs in mixed tasks will be further studied.

## Figures and Tables

**Table 1:** Initialization information of platforms and UAVs.

Platform	Location	UAV	Cruising Speed (km/h)	Maximum Range (km)
$S_{0}$	[19.37, 37.39]	$U_{0}$	0.22	1000
		$U_{1}$	0.18	1200
$S_{1}$	[65.77, 57.97]	$U_{2}$	0.16	1500
		$U_{3}$	0.22	1000
$S_{2}$	[42.48, 21.43]	$U_{4}$	0.18	1200
		$U_{5}$	0.16	1500
$S_{3}$	[32.59, 75.64]	$U_{6}$	0.22	1000
		$U_{7}$	0.30	800

**Table 2:** Initialization information of targets.

Target	Location	Radius of Circular Route	Time Window
$T_{0}$	[218.78, 270.38]	2.37	
$T_{1}$	[195.39, 162.48]	1.67	[900, 1200]
$T_{2}$	[146.44, 239.28]	2.18	
$T_{3}$	[156.53, 289.27]	2.01	
$T_{4}$	[184.89, 215.84]	1.05	[1300, 1800]
$T_{5}$	[199.31, 250.61]	2.78	
$T_{6}$	[231.33, 110.05]	1.64	
$T_{7}$	[227.74, 154.97]	1.97	
$T_{8}$	[126.38, 294.10]	2.33	
$T_{9}$	[109.69, 185.35]	1.03	
$T_{10}$	[280.76, 266.99]	1.31	[800, 1300]
$T_{11}$	[260.75, 287.63]	2.22	
$T_{12}$	[275.67, 125.95]	1.23	
$T_{13}$	[173.67, 250.11]	2.47	
$T_{14}$	[193.87, 108.35]	1.96	[1000, 1600]
$T_{15}$	[268.56, 107.26]	2.21	
$T_{16}$	[254.66, 183.98]	2.02	
$T_{17}$	[260.90, 226.18]	1.31	
$T_{18}$	[290.79, 172.73]	2.32	[1400, 1800]
$T_{19}$	[125.60, 254.88]	2.11	

**Table 3:** Data of new targets.

Target	Location	Radius of Circular Route	Time Window
$T_{n}^{1}$	[280.63, 278.70]	2.00	
$T_{n}^{2}$	[180.89, 173.82]	1.03	
$T_{n}^{3}$	[151.43, 198.77]	2.85	[1400, 1700]
$T_{n}^{4}$	[215.02, 172.35]	2.06	
$T_{n}^{5}$	[191.46, 292.98]	1.62	
$T_{n}^{6}$	[251.27, 263.65]	2.46	
$T_{n}^{7}$	[257.51, 280.33]	2.44	
$T_{n}^{8}$	[173.28, 290.10]	1.18	[1600, 1900]
$T_{n}^{9}$	[296.40, 146.77]	1.78	
$T_{n}^{10}$	[275.39, 207.76]	1.30	
$T_{n}^{11}$	[296.49, 161.63]	1.25	
$T_{n}^{12}$	[223.44, 166.88]	2.36	[1500, 1900]
$T_{n}^{13}$	[143.55, 265.13]	2.41	
$T_{n}^{14}$	[179.71, 228.92]	2.31	

**Table 4:** Statistics of communication times in test 5.

Damaged UAV	$TL_{un}$	$n_{d}$	$n_{un}$	Communication Times	
				CNP-Based	TS-DTA
$U_{0}$	[2, 13, 5, 4]	5	4	32	22
$U_{1}$	[6, 15, 12]	5	3	24	12
$U_{3}$	[16, 17, 18]	5	3	24	18
$U_{6}$	[8, 19, 9]	5	3	24	12
$U_{0},U_{1}$	[2, 13, 5, 4, 6, 15, 12]	4	7	42	19
$U_{0},U_{3}$	[2, 13, 5, 4, 16, 17, 18]	4	7	42	26
$U_{0},U_{6}$	[2, 13, 5, 4, 8, 19, 9]	4	7	42	17

**Table 5:** Statistics of communication times in test 6.

New Target	$n_{d}$	$n_{un}$	Times of Communication	
			CNP-Based	TS-DTA
$T_{n}^{1},T_{n}^{2}$	6	2	20	14
$T_{n}^{1},T_{n}^{2},T_{n}^{3},T_{n}^{4}$	6	4	40	21
$T_{n}^{1},T_{n}^{2},T_{n}^{3},T_{n}^{4},T_{n}^{5},T_{n}^{6}$	6	6	60	21
$T_{n}^{1},T_{n}^{2},T_{n}^{3},T_{n}^{4},T_{n}^{5},T_{n}^{6},T_{n}^{7},T_{n}^{8}$	6	8	80	28
$T_{n}^{1},T_{n}^{2},T_{n}^{3},T_{n}^{4},T_{n}^{5}$ $T_{n}^{6},T_{n}^{7},T_{n}^{8},T_{n}^{9},T_{n}^{10}$	6	10	100	28
$T_{n}^{1},T_{n}^{2},T_{n}^{3},T_{n}^{4},T_{n}^{5},T_{n}^{6}$ $T_{n}^{7},T_{n}^{8},T_{n}^{9},T_{n}^{10},T_{n}^{11},T_{n}^{12}$	6	12	120	35
$T_{n}^{1},T_{n}^{2},T_{n}^{3},T_{n}^{4},T_{n}^{5},T_{n}^{6},T_{n}^{7}$ $T_{n}^{8},T_{n}^{9},T_{n}^{10},T_{n}^{11},T_{n}^{12},T_{n}^{13},T_{n}^{14},$	6	14	140	39

**Table 6:** Statistics of communication times in test 7.

Damaged UAV	New Target	$n_{d}$	$n_{un}$	Times of Communication	
				CNP-Based	TS-DTA
$U_{0}$	$T_{n}^{1},T_{n}^{2}$	5	5	40	18
$U_{1}$	$T_{n}^{1},T_{n}^{2},T_{n}^{3},T_{n}^{4},T_{n}^{5},T_{n}^{6}$	5	9	72	22
$U_{2}$	$T_{n}^{1},T_{n}^{2},T_{n}^{3},T_{n}^{4},T_{n}^{5},T_{n}^{6},T_{n}^{7},T_{n}^{8}$	5	10	72	30
$U_{0},U_{2}$	$T_{n}^{9},T_{n}^{10},T_{n}^{11},T_{n}^{12}$	4	9	48	23
$U_{0},U_{7}$	$T_{n}^{9},T_{n}^{10},T_{n}^{11},T_{n}^{12},T_{n}^{13},T_{n}^{14}$	4	11	66	20

**Table 7:** The CPU running time and the overall cost of UAV formation.

$n_{un}$	Indicator	RPSO $n_{p}\;$ = 100	RPSO $n_{p}$ = 300	RPSO $n_{p}$ = 500	IEPPSO $n_{p}$ = 100	IEPPSO $n_{p}$ = 300	IEPPSO $n_{p}$ = 500	CNP-Based	TS-DTA
2	$t_{cpu}$	1.60	5.12	8.57	11.32	28.99	49.94	0.016	0.016
	fc	2889.13	2208.32	2200.23	2047.23	2032.55	2001.19	2008.65	2016.87
4	$t_{cpu}$	1.72	5.34	9.11	16.79	42.01	64.97	0.031	0.016
	fc	3124.00	2389.12	2349.33	2116.75	2086.81	2071.35	2090.08	2160.61
6	$t_{cpu}$	1.83	5.62	9.30	27.57	62.81	96.75	0.031	0.031
	fc	4747.52	3134.25	2618.54	2264.27	2162.50	2178.09	2216.05	2216.05
8	$t_{cpu}$	1.91	5.90	9.95	41.08	87.14	129.78	0.047	0.047
	fc	6868.40	3960.38	3785.01	2357.67	2271.90	2260.06	2346.45	2348.27
10	$t_{cpu}$	1.98	6.25	10.36	67.69	138.86	191.72	0.031	0.047
	fc	8484.40	3610.04	3567.31	2444.06	2323.40	2282.11	2355.24	2355.34

## Data Availability

Data are contained within the article.
